# Red Cell Distribution Width Is Associated with Presence, Stage, and Grade in Patients with Renal Cell Carcinoma

**DOI:** 10.1155/2014/860419

**Published:** 2014-12-16

**Authors:** Fang-Ming Wang, Gongjun Xu, Yan Zhang, Lu-Lin Ma

**Affiliations:** ^1^Department of Urology, Peking University Third Hospital, 49 North Garden Road, Beijing 100191, China; ^2^School of Statistics, University of Minnesota, Minneapolis, MN 55455, USA; ^3^Division of Dyslipidemia, State Key Laboratory of Cardiovascular Disease, Fu Wai Hospital, National Center for Cardiovascular Disease, Chinese Academy of Medical Sciences and Peking Union Medical College, Beijing 100037, China

## Abstract

It has been reported that red blood cell width (RDW) is a marker associated with the presence and adverse outcomes of various diseases. However, no data are available on the correlation of RDW with presence, stage, and grade in patients with renal cell carcinoma (RCC) yet. By retrospectively analyzing clinical and laboratory data at baseline of histologically confirmed RCC cases and controls, the present study demonstrated that the RDW values were significantly higher in patients with RCC than those in controls, and the baseline RDW value was independently associated with the presence of RCC. Besides, the data revealed a positive association between RCC stage and grade and the level of RDW. These findings may have important clinical implications due to future application using a RDW value in predicting RCC.

## 1. Introduction

Renal cell carcinoma (RCC) is the most frequent malignant tumor of kidney with a rising incidence of 60920 patients and 13120 cancer-related deaths in the USA in 2011 [1]. Over the past decades, the incidence of RCC has been increasing worldwide. The increase in disease rates, together with the fact that no diagnostic marker is available, has high socioeconomic effects [2]. Therefore, inexpensive and convenient markers which could be used in the prediction of RCC would be desirable.

Red cell distribution width (RDW) is a measure of size variability in circulating red blood cells and is routinely reported as a part of complete blood count analysis [3]. Its main clinical application has been limited to the differential diagnosis of anemia [4]. Recent studies have reported the association between high RDW levels and increased mortality in patients with cardiovascular disease [5–9], brain vascular disease [10], strokes [11], septicemia [12], chronic obstructive pulmonary disease [13], and hepatitis B [14]. Elevated RDW values were also shown to be associated with increased risk of mortality in the general population [15–17].

There are few reports on the relationship between RDW and malignant tumors. It has been reported that RDW was significantly higher in patients with breast cancer, compared with patients with fibroadenomas [18]. Besides, several studies have reported that RDW could distinguish malignant from benign tumors or predict the presence of malignant tumors [19–21]. Moreover, a recent study revealed that RDW is associated with cancer stage and survival in lung cancer patients [22]. The mechanism underlying associations of RDW with the above diseases has not been elucidated, but high levels of RDW are thought to be provoked by chronic inflammation, poor nutritional status, and changes in erythropoiesis [16, 23]. Thus, we speculated that RDW values might be associated with RCC, which is known to evoke chronic inflammation and malnutrition [24, 25]. However, there is no specific study assessing the relationship of RDW with clinical and pathological parameters of RCC.

Therefore, in this study, we retrospectively evaluated whether RDW has a potential role in predicting the presence of RCC and further examined the associations between RDW and RCC stage and grade.

## 2. Methods

### 2.1. Study Design and Population

The study complied with the Declaration of Helsinki and was approved by our Institute Ethical Committee. All subject names, initials, or hospital numbers were not used in the text, table, or illustrative materials of this study.

A retrospective analysis was conducted in patients with primary diagnosed, pathologically confirmed, and sporadic RCC and controls identified hospital patients with simple renal cyst, between January 2010 and June 2013 at Department of Urology at Peking University Third Hospital. The exclusion criteria of the study were the presence of medical history of other malignancy, pregnancy, kidney transplantation, hematological disorders, severe anemia, infectious or inflammatory disease, iron supplementation therapy, recent venous thrombosis (past 6 months), recent blood transfusion (past 3 months), chronic obstructive pulmonary disease, hepatitis B or C, heart failure, arrhythmia, untreated thyroid disease, and severe liver and/or renal insufficiency as described previously [26]. All data on age, gender, body mass index (BMI), history of hypertension or diabetes, smoking, blood parameters, histology, stage at diagnosis (2009 AJCC TNM classification), and Fuhrman grading were obtained from electronic records and medical charts.

### 2.2. Biomarker Measurements

Venous blood samples were obtained from each patient at baseline upon admission. The RDW value, hemoglobin (HB), mean cell volume (MCV), platelet, and white blood cell (WBC) were determined using an automated blood cell counter with an automated hematology analyzer XE-2100 (Sysmex Corporation, Kobe, Japan). The normal range for RDW in general and in our laboratory is 11% to 15%. The level of albumin was measured using Olympus AU2700 Analyzer (Olympus, Tokyo, Japan). The Westergren method was used for the measurement of erythrocyte sedimentation rate (ESR).

### 2.3. Exposure Definition

Risk factor definitions were as follows. (1) BMI was defined as the first reported weight (in kilograms) divided by height in square meters, and BMI ≥25 kg/m^2^ was considered as overweight; (2) the threshold of hypertension was set at 140 and 90 mmHg for systolic and diastolic blood pressure, respectively, on three consecutive occasions; (3) diabetes was based on either one of the following criteria: fasting serum glucose level ≥7.0 mmol/L, normal fasting serum glucose level owing to usage of antidiabetic medication, or self-report of a physician's diagnosis of diabetes; (4) smoking meant current smokers or those who had ever smoked at least 100 cigarettes per year [27, 28].

### 2.4. Statistical Analyses

Quantitative variables were expressed as mean ± standard deviation (SD), and qualitative variables were expressed as numbers and percentages. Continuous variables and categorical variables were analyzed by the Student's *t*-tests or chi-squared statistic tests when appropriate. The univariate and multivariate logistic regression analysis were used to estimate odds ratios (OR) and 95% confidence intervals (CI), including RDW, other blood parameters, and previously identified clinical variables, such as age, gender, BMI, history of hypertension or diabetes, and smoking. Receivers operating characteristic (ROC) curves were constructed at the most discriminating cut-off point values aiming at documenting the predictive power of RDW for the presence of RCC and advanced RCC (stages 3 and 4). Simple linear regression analysis was performed to explore the association of RDW with RCC stage and grade. Spearman test was used to observe the correlation between RDW and other variables in RCC patients. The values of *P* were two-sided for all statistical tests. A value of *P* < 0.05 was considered statistically significant. SPSS program (version 19.0, SPSS, Chicago, IL, USA) was used for statistical analyses.

## 3. Results

### 3.1. Characteristics of the Cases and Controls

The study included 318 newly diagnosed sporadic RCC cases (age range: 13–83 years, average age: 56.83 years) and 238 controls (age range: 20–80 years, average age: 55.10 years). Distribution of RCC cases and controls according to clinical and laboratory characteristics is summarized in Table 1. In brief, no significant differences in age, gender, hypertension, and diabetes were observed between groups. More than 50% of cases were overweight. RDW levels of cases were significantly higher than that of controls (13.27 ± 0.90 versus 12.88 ± 0.51, *P* < 0.001). In addition, patients with RCC showed lower HB, MCV, and albumin levels but higher smoking rate, platelet, WBC, and ESR levels. Clear renal cell carcinoma cases were mostly (282/318, 88.68%) the conventional cell type, the most common of which were Grade II tumors (45.74%). Pathologic T1 (pT1) tumors account the most (*n* = 239, 75.16%) in tumor classification.

### 3.2. RDW and Other Parameters for the Risk of RCC

**Figure 1:**
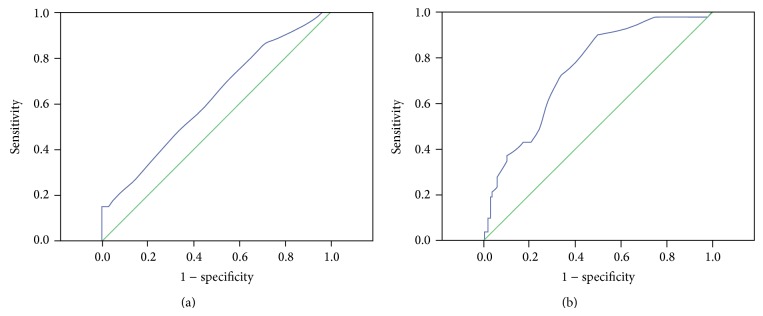
The results of receiver operating characteristic (ROC) curve analysis for the predictive power of RDW in predicting presence of RCC (a) and advanced RCC (b).

As shown in Table 2, parameters including smoking, RDW, HB, MCV, albumin, WBC, platelet, and ESR found to be statistically significant in univariate analyses were entered into multivariate logistic regression analysis. The data indicated that RDW, WBC, albumin, ESR, and smoking were independently correlated with the presence of RCC in multivariate logistic regression analysis. In particular, RDW was proved to be an independent predictor for presence of RCC after adjusting for the known confounders (OR = 1.808, 95% CI 1.296–2.523, and *P* < 0.001). Area under ROC curve (AUC) of RDW was 0.624 (95% CI 0.578–0.670, *P* < 0.001) for predicting RCC (Figure 1(a)). The optimal cut-off value of RDW to predict the presence of RCC was 12.85% (sensitivity of 65.09% and specificity of 51.50%).

### 3.3. RDW and RCC Stage

**Figure 2:**
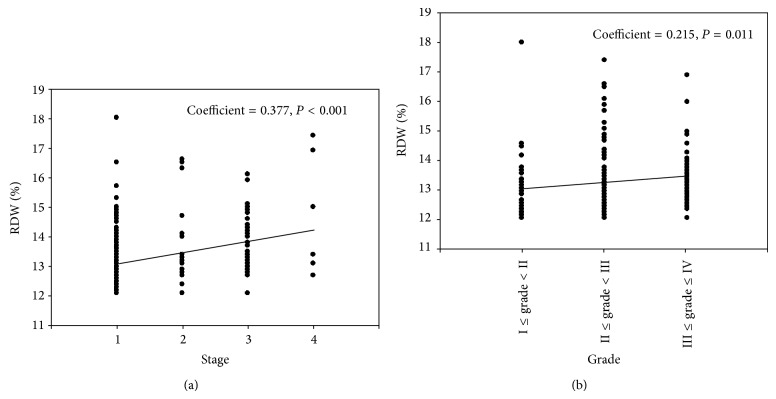
Scatter plot diagram showing the positive correlation of cancer stage (a) and grade (b) with RDW level.

RDW values in RCC patients according to cancer stages were examined using simple linear regression analysis, and we found a positive association between cancer stage and RDW value (coefficient = 0.377 and *P* < 0.001, Figure 2(a)). There is an evident trend that RDW value increased with the progression of cancer stage. AUC of RDW was 0.75 (95% CI 0.683–0.818, *P* < 0.001) for predicting the presence of advanced RCC (Figure 1(b)). The optimal cut-off value of RDW to predict advanced RCC was 13.15% (sensitivity of 76.47% and specificity of 61.05%).

### 3.4. RDW and Grade of Clear Cell Carcinoma

RDW values in clear cell carcinoma patients according to Fuhrman grading system were evaluated by simple linear regression analysis. There existed a positive association between cancer grade and RDW value (coefficient = 0.215 and *P* = 0.011, Figure 2(b)).

### 3.5. Correlations of RDW

To explore the relationships of RDW with other parameters in patients with RCC, Spearman correlation evaluation was performed in the present study. As shown in Table 3, RDW showed a significant inverse correlation with BMI, HB, MCV, and albumin (*r* = −0.186, −0.306, −0.164, and −0.262, resp., all *P* ≤ 0.003), and a significant positive correlation with age, platelet, WBC and ESR. (*r* = 0.256, 0.174, 0.149, and 0.155, resp., all *P* < 0.01). However, there was no correlation of RDW with gender, smoking, hypertension, or diabetes mellitus.

## 4. Discussion

To our knowledge, the present study is the first to analyze RDW in RCC patients. We demonstrated that the RDW values were significantly higher in patients with RCC than those in controls, and the baseline RDW value was independently associated with the presence of RCC. Furthermore, the ROC curve indicated that high RDW value (12.85%) could predict the presence of RCC. In addition, the data revealed a positive association between RCC stage, grade, and the level of RDW and also determined the cut-off points (13.15%) of RDW which can be valuable for predicting advanced RCC. Our findings may have important clinical implications due to future application using a RDW value in predicting RCC.

RDW reflects the variability in circulating RBC size. It is based on the width of the RBC volume distribution curve, with larger values indicating greater variability [29]. RDW is elevated when there is increased red cell destruction, or, more commonly, ineffective red cell production. RDW may represent nutritional deficiency (e.g., iron, vitamin B12, or folic acid), bone marrow depression, or chronic inflammation [30–32]. These conditions are more or less prevalent in cancers. However, extremely limited data exist reporting the association between RDW and cancers. In a study by Baicus et al., RDW was significantly elevated in a cohort of patients with various types of malignancies, when compared to noncancer patients [20]. Spell et al. [21] demonstrated RDW can help better identify those patients with colon cancer. Moreover, a recent study conducted by Beyazit et al. [19] indicated that elevated RDW could be a useful biomarker in order to discriminate benign from malignant causes of biliary obstruction, with a sensitivity of 72% and specificity of 69%, using 14.8% as a cut-off value for RDW. Seretis et al. [18] found that RDW was significantly higher in patients with breast cancer than those with fibroadenomas. Nevertheless, none of the above studies referred to the relationship of RDW with RCC, let alone stage and grade of RCC.

In the current study, we extended previous studies and for the first time found that patients with RCC had significantly higher RDW values and baseline RDW levels remain an independent predictor for patients with RCC using univariate and multivariate logistic regression analysis. Furthermore, our study revealed a positive association between clinical RCC stage and the levels of RDW. The trend is evident that RDW level increased with the progression of cancer stage. Besides, according to the ROC curve, RDW might be a proper marker to predict the progression of RCC. Similarly, a recent retrospective study [22] also demonstrated a positive association of RDW levels with cancer stage in patients with lung cancer. Another interesting finding of our study was the fact that RDW was positively associated with the Fuhrman grade in clear cell carcinoma in the general trend; this particular finding was in accordance with the rationale of elevation of RDW according to the presence of a more active inflammatory process, as a higher tumor grade generally enhances the local and systematic inflammatory reaction [33]. However, Seretis et al. [18] reported that RDW was inversely associated with the tumor grade in patients with breast cancer; our findings are inconsistent with theirs, which can be explained as the different biological activity and inflammatory reaction of different tumors.

The exact mechanisms of correlation between higher values of RDW and RCC are somewhat unclear. One very likely mechanism is inflammation. It has been recognized that cancer progression depends on a complex interaction of the tumor and host inflammatory response [34–36]. In fact, recent findings have suggested that inflammatory cytokines hs-CRP, IL-6, and other proinflammatory cytokines play pivotal roles in RCC [37–39]. A strong association between RDW and inflammatory markers was found in a large cohort of unselected adult outpatients, as well as patients with inflammatory bowel disease [40, 41]. Inflammation might contribute to increased RDW values not only by impairing iron metabolism but also by inhibiting the production of or response to erythropoietin or by reducing erythrocyte lifespan [41]. Besides that, according to Bion [42], RDW may reflect the extent of the patient's physiological reserve, when the reserve is reduced or already exhausted in disease situation, anisocytotic, immature red cells appear in the circulation, which results in an elevated RDW. Although this reserve theory was evaluated in acute illnesses, patients with a chronic disease, such as RCC, might also have disparities in physiological reserve. In the present study, the correlation analysis indicated that patients with higher values of RDW tended to have lower levels of hemoglobin and serum albumin and higher WBC and ESR, which strengthened the hypothesis that inflammation and malnutrition may be involved in higher RDW levels in patients with RCC.

Nonetheless, there were several limitations of present study. Firstly, the sample size is relatively small and the findings could be from chance. Secondly, this is an observational study and so still there could be residual confounding factors. Finally, we did not evaluate the prognostic value of RDW in our population. Further study is needed to examine the role of the RDW in predicting the clinical outcomes in a large sample size and long-term follow-up.

## 5. Conclusions

Summarily, the present study revealed the association between higher RDW values and increased risk of RCC. This association was not affected by adjustment for other known risk factors. Additionally, the RDW values were positively associated with cancer stage in RCC and grade in clear cell carcinoma. Because RDW values can be routinely examined by complete blood count tests, it might be an easily available predictor for RCC and its stage and grade.

## Figures and Tables

**Table 1:** Description and comparison of clinical and laboratory characteristics of the study subjects.

Variables	RCC (*n* = 318)	Controls (*n* = 238)	*P* value
Age (years)	56.83 ± 11.68	55.10 ± 13.90	0.122
Gender (male/female)	210/108	146/92	0.284
BMI (*n* (%))			0.370
<25	152 (47.80)	124 (52.10)	
≥25	166 (52.20)	114 (47.90)	
Hypertension (*n* (%))	120 (37.74)	86 (36.13)	0.723
Diabetes mellitus (*n* (%))	46 (14.47)	23 (9.66)	0.093
Smoking (*n* (%))	63 (19.81)	29 (12.18)	**0.021**
RDW (%)	13.27 ± 0.90	12.88 ± 0.51	**<0.001 **
HB (g/L)	138.83 ± 15.53	143.02 ± 13.69	**0.001**
MCV (fL)	92.05 ± 4.79	92.95 ± 3.77	**0.013**
Platelet (10^9^/L)	214.04 ± 67.83	198.26 ± 48.75	**0.001**
WBC (10^9^/L)	6.51 ± 1.74	5.72 ± 1.32	**<0.001 **
Albumin (g/L)	43.00 ± 4.28	44.37 ± 3.14	**<0.001 **
ESR (mm/h)	11.00 (5.00–19.00)	6.00 (3.00–10.00)	**<0.001 **
Side (*n* (%))			
Left-sided	135 (42.45)	131 (55.04)	
Right-sided	176 (55.35)	96 (40.34)	
Two-sided	7 (2.20)	11 (4.62)	
Pathologic type (*n* (%))			
Clear cell carcinoma	282 (88.68)		
Papillary renal cell carcinoma	15 (4.72)		
Chromophobe renal cell carcinoma	11 (3. 46)		
Others	10 (3.14)		
Stage at diagnosis (*n* (%))			
T1	239 (75.16)		
T2	28 (8.81)		
T3	45 (14.15)		
T4	6 (1.89)		
M1	20 (6.29)		
M0	298 (93.71)		
Fuhrman grading of clear cell carcinoma (*n* (%))			
Grade I	22 (7.80)		
Grades I-II	35 (12.41)		
Grade II	129 (45.74)		
Grades II-III	41 (14.54)		
Grade III	47 (16.67)		
Grades III-IV	7 (2.48)		
Grade IV	1 (0.35)		

Data are expressed as *n* (%), median (IQR), or mean ± SD. BMI: body mass index; RDW: red cell distribution width; HB: hemoglobin; MCV: mean cell volume; WBC: white blood cell; ESR: erythrocyte sedimentation rate.

**Table 2:** Logistic regression analysis of coexistence of parameters and RCC risk.

Parameters	Univariate analysis		Multivariate analysis	
	OR (95% CI)	*P* value	OR (95% CI)	*P* value
Age	1.011 (0.997–1.024)	0.113		
Gender	1.225 (0.864–1.737)	0.254		
BMI	1.051 (0.996–1.109)	0.069		
Hypertension	1.071 (0.756–1.518)	0.699		
Diabetes	1.581 (0.929–2.690)	0.091		
Smoking	1.781 (1.106–2.867)	**0.018**	1.944 (1.104–3.423)	**0.021**
RDW	2.326 (1.728–3.132)	**<0.001**	1.808 (1.296–2.523)	**<0.001**
HB	0.981 (0.969–0.992)	**0.001**	0.987 (0.971–1.004)	0.129
MCV	0.953 (0.915–0.991)	**0.017**	0.962 (0.915–1.012)	0.138
Platelet	1.005 (1.002–1.007)	**0.003**	0.999 (0.995–1.003)	0.653
WBC	1.406 (1.245–1.588)	**<0.001**	1.355 (1.161–1.581)	**<0.001**
Albumin	0.908 (0.865–0.953)	**<0.001**	0.926 (0.872–0.983)	**0.012**
ESR	1.111 (1.077–1.146)	**<0.001**	1.092 (1.062–1.122)	**<0.001**

RDW: red cell distribution width; HB: hemoglobin; MCV: mean cell volume; WBC: white blood cell; ESR: erythrocyte sedimentation rate.

**Table 3:** Correlations of various parameters with RDW in RCC patients.

Parameters	*r*	*P*
Age	0.256	**<0.001**
Gender	−0.076	0.177
BMI	−0.186	**0.002**
Hypertension	0.04	0.479
Diabetes mellitus	0.023	0.682
Smoking	0.073	0.194
HB	−0.306	**<0.001**
MCV	−0.164	**0.003**
Platelet	0.174	**0.002**
WBC	0.149	**0.008**
Albumin	−0.262	**<0.001**
ESR	0.155	**<0.001**

BMI: body mass index; HB: hemoglobin; MCV: mean cell volume; WBC: white blood cell; ESR: erythrocyte sedimentation rate.
